# Geoengineering as Collective Experimentation

**DOI:** 10.1007/s11948-015-9646-0

**Published:** 2015-04-11

**Authors:** Jack Stilgoe

**Affiliations:** University College London, Gower Street, London, WC1E 6BT UK

**Keywords:** Geoengineering, Climate engineering, Governance, Responsible research and innovation, Collective experimentation

## Abstract

Geoengineering is defined as the ‘deliberate and large-scale intervention in the Earth’s climatic system with the aim of reducing global warming’. The technological proposals for doing this are highly speculative. Research is at an early stage, but there is a strong consensus that technologies would, if realisable, have profound and surprising ramifications. Geoengineering would seem to be an archetype of technology as social experiment, blurring lines that separate research from deployment and scientific knowledge from technological artefacts. Looking into the experimental systems of geoengineering, we can see the negotiation of what is known and unknown. The paper argues that, in renegotiating such systems, we can approach a new mode of governance—collective experimentation. This has important ramifications not just for how we imagine future geoengineering technologies, but also for how we govern geoengineering experiments currently under discussion.

## Introduction

In September 2011, a proposed experiment was announced by a group of British University scientists that, from one perspective, seemed mundane. The idea was to float a tethered helium balloon a kilometre up in the sky with a hose attached. A pump would deliver a few dozen litres of water to the top of the hose, where it would emerge as a mist, evaporating before it hit the ground. At the time, there was a strong consensus among the scientists involved, their universities and outside observers that the experiment was not particularly risky, nor did it run against established ethical protocols. The experiment, however, became a condensation point for controversy because it was also, to use the researchers’ own words, *‘the first field test of a geoengineering technology in the UK’* (see Stilgoe 2015 for a fuller account). The experiment was part of a project called SPICE—Stratospheric Particle Injection for Climate Engineering. In the end, the experiment never got off the ground, figuratively or physically. Citing their own concerns about intellectual property and the governance of geoengineering, the researchers called it off. Nevertheless, the controversy generated provides an important entry point for ‘informal technology assessment’ (Rip 1987) of geoengineering as a social experiment (Stilgoe et al. 2013).

Geoengineering (or climate engineering) encompasses a set of ideas for technological fixes to global climate change. The range of proposals is large, but in the main they concentrate either on removing carbon dioxide from the atmosphere or on cutting the amount of sunlight that reaches the surface of the Earth. The former category includes schemes to massively expand forests or seed oceans in order to encourage algae, as well as machines for capturing carbon directly from the air. The latter ranges from sunshades positioned in space between the Earth and Sun to the whitening of roofs on buildings. Within this category of so-called Solar Radiation Management, the idea of stratospheric particle injection—creating a reflective haze in the Earth’s stratosphere—has attracted most attention because early assessments suggest that it has the greatest potential to reduce incoming sunlight while being (relatively) affordable. David Keith, currently the world’s most prominent geoengineering researcher, opens his recent book—*A case for climate engineering—*by claiming with some certainty that,

It is possible to cool the planet by injecting reflective particles of sulfuric acid into the upper atmosphere where they would scatter a tiny fraction of incoming sunlight back to space, creating a thin sunshade for the ground beneath. To say that it’s “possible” understates the case: it is cheap and technically easy. (Keith 2013, p. ix)

As i will describe below, there are plenty of reasons to question the desirability of geoengineering. David Keith would join the majority of scientists in the nascent domain of geoengineering research who would doubt whether geoengineering was a Good Idea, although most would not share Keith’s level of conviction. Proposals for geoengineering, which began as an extension of cold war technocratic modernism (see Fleming 2010), have, with their 21st century re-emergence, taken on a reflexive flavour (cf Beck 1992). Nevertheless, geoengineering has, despite myriad uncertainties about its doability and desirability, rapidly acquired a deterministic frame, based on the assumption that it is ‘cheap’ and ‘easy’. Following the pattern of what Joly and colleagues (2010) call the ‘economics of techno-scientific promises’, geoengineering has been naturalised by its researchers, treated as a thing in the world to be understood rather than a highly controversial, highly speculative set of technological fix proposals.

In this paper, I argue that the governance debate surrounding geoengineering can benefit from a view that starts with recognition of the social experimental nature of emerging technologies. The field of geoengineering research is small but growing. The uncertainties are vast and the likelihoods of predictability and control are tiny. Geoengineering would, as currently imagined, seem to represent an archetypical experimental technology. But we should not presume to know what geoengineering technologies will look like, if they are indeed realised. It is therefore also important to look at the experiments taking place within geoengineering research, experiments in which future geoengineering technologies and imaginaries (Jasanoff, in press) are being shaped. The emerging technology of geoengineering represents an experimental system in which knowns and unknowns are negotiated, in public discourse and in research projects. As with SPICE, the potential for reframing experimental means and ends suggests the possibility of a new mode of governance, one of collective experimentation, with implications for how we think about other geoengineering research experiments.

## Responsible Research and Innovation

The emergence of geoengineering as a research agenda and a ‘matter of concern’ (Latour 2004) has coincided with growing US and European interest in ‘responsible research and innovation’, (RRI) ‘responsible innovation’ or the ‘responsible development’ of new technologies. There has been some institutional uptake of these terms, and possibly the ideas that they carry, within the European Commission, the UK Research Councils and the National Science Foundation respectively.^1^ Although institutions may neglect, wilfully or otherwise, to mention it, these terms have their roots in debates about the possibilities of broadening the basis for technology assessment (Guston and Sarewitz 2002; Rip et al. 1995), reinvigorating the politics of technology (Winner 1980) and aligning science and innovation with social needs. While there have been substantial policy efforts in some countries to ‘open up’ (Stirling 2008) public debates about emerging technologies, these have typically been disconnected from any policy or scientific response. RRI offers the possibility of shifting governance debates away from problematising publics to focus on research and innovation themselves. (Pellizoni 2004) suggests that we should pay more attention to the limits to responsiveness. In doing so, he reconnects debates about responsibility to an older discussion of the social control of technology. David Collingridge (1980) described the dilemma of control in these terms:

[A]ttempting to control a technology is difficult, and not rarely impossible, because during its early stages, when it can be controlled, not enough can be known about its harmful social consequences to warrant controlling its development; but by the time these consequences are apparent, control has become costly and slow. (Collingridge 1980, p 19)

As Liebert and Schmidt (2010) point out, Collingridge is better remembered for describing this dilemma than for his normative aim of finding ways to govern despite it. Collingridge was interested in identifying and seeking to ameliorate ‘the roots of inflexibility’ (Collingridge 1980, p. 45). We therefore need not be fatalistic, not least because, as Liebert and Schmidt go on to conclude, technologies may not be ‘controlled’ according to particular decisions in the light of particular knowledge in the formal way that Collingridge seems at first to assume and then goes on to himself critique. Technologies, as later constructivist studies would conclude, are as much a result of unquestioned assumptions or implicit values (see Williams and Edge (1996) for a summary). The attempt to govern despite the impossibility of prediction has acquired the term ‘anticipatory governance’ (see Guston (2014) and Nordmann (2014) for a recent discussion).

Nor should we see uncertainty and ignorance as essential and problematic properties of technology. Uncertainty is constructed in scientific and innovative practice and attempts are made to exert both technical and social control over its bounds (Jasanoff and Wynne 1998). In public issues, uncertainties can be coproduced and reproduced as public concerns are interpreted, legitimised or rejected (Stilgoe 2007). As I will describe, the construction of experimental systems therefore plays a crucial political role by giving meaning to particular uncertainties.

From his re-reading of the Green Revolution, Collingridge demands that we pay closer attention to the contestation of problems to which technologies are offered as solutions (see Morozov (2013) for a recent popular account of the similar dynamics in what he calls the ‘solutionism’ of digital technologies). Discourses of responsible research and innovation attempt, in the face of what are perceived as growing pressures towards neoliberal science (Lave et al. 2010; Pellizoni and Ylönen 2012), to draw stronger links with global societal challenges (von Schomberg 2012). But doing so introduces a profound question of democratisation. Technology, following Winner (1977), is itself a powerful form of legislation. If problems are constructed in order to serve particular solutions, rather than the other way around, then an important task of responsible research and innovation should surely be one of reflexivity on problem definition. Science and technology may themselves not hold the single or best answer, and may crowd out alternative approaches of social innovation.

What, then, does it mean to ‘care’ for the futures to which science and innovation contribute (Groves 2014; Owen et al. 2013; Stilgoe 2015)? First, the idea of care seems more satisfactory than ‘control’, the term used by Collingridge. Just as we recognise that the unintended consequences of technology are not completely predictable or controllable (Wynne 1988), so we should recognise that the trajectories of technology cannot themselves be predicted and controlled (see Stirling 2014). A care-ful approach is less likely to involve prohibition (Marchant and Pope 2009) than what Kuhlmann and colleagues (2012) call ‘tentative governance’, encompassing “provisional, flexible, revisable, dynamic and open approaches that include experimentation, learning, reflexivity, and reversibility”. This is, incidentally, close to David Collingridge’s prescription of ‘corrigibility’.

Scientists, innovators and others may argue that they are taking responsibility not just through conventional mechanisms of research integrity but also by engaging in what Alfred Nordmann (2007) has labelled ‘speculative ethics’. Certainly, geoengineering research has seen more than its fair share of speculative ethics, which, by asking what happens ‘if’ geoengineering futures are realised, contributes to a narrative of inevitability (Stilgoe 2015). Speculative ethics has joined risk assessment as part of an attempt to make techno-scientific promises of innovation more explicitly ‘responsible’, but they risk closing down decision making rather than opening it up to new possibilities. The dominant governance discourse tends towards ‘containment’ (Jasanoff and Kim 2009) of not just risk and ethics, but also of public debate. Peter-Paul Verbeek (2010) makes the case for reconnecting the empirical and ethical strands of the philosophy of technology to move from a mode of ‘technology assessment’ to one of ‘technology accompaniment’. In this latter mode, the imaginaries of geoengineering, which embed particular understandings of problems and solutions, can be adequately interrogated.

As I will argue, the impossibility of control in a scientific sense, let alone as a public issue, would put geoengineering alongside technologies such as genetically modified crops (Levidow and Carr 2007) and nuclear energy (Krohn and Weingart 1987), whose testing and deployment can be constructively seen as forms of social experiment (Krohn and Weyer 1994). This line of academic study builds upon, informs and is informed by a critical discourse about technology from commentators and NGOs that uses the language of experimentation to argue that technologies are less predictable, less well-understood and less controllable than their proponents would have us believe. The political writer John Gray (2004) expresses anguish that, ‘The world today is a vast unsupervised laboratory, in which a multitude of experiments are simultaneously underway. Many of these experiments are not recognised as such’.

Bonneuil and colleagues argue that we should not look to the inherent riskiness of open-air experimentation but instead look to experiments as a site for the contestation of the politics of emerging technologies. They describe how, in France, field experiments with genetically modified crops were reframed through public controversy. In the decade up to 1996, thousands of field experiments took place without arousing wider interest. Over the next decade, these experiments became the focus of a debate less about the health and environmental risks of a particular technique than about the future economics and politics of agriculture. Experiments that had previously been ‘entrenched’ as being routine scientific affairs, and shielded from public view, were dramatically reinterpreted as incursions into the social arena. Activists targeted and destroyed crop trials, justifying this as both a means to an end (attracting public attention) and an end in itself (preventing what they regarded as genetic contamination) (Bonneuil et al. 2008).

If we recognise the experimental nature of emerging technology from the start, we can put questions of democracy back into governance, asking how scientists and others should negotiate ‘the conditions for the performance of experiments in and on society’ (Krohn and Weyer 1994, p. 181). The ethical questions expand beyond consideration of the ethical ‘implications’ of technology to also include experimental care and ethics, which prompts consideration of who the participants are and the extent of their informed consent. The democratic governance of innovation therefore means asking what counts as legitimate experimentation (van de Poel (this issue)), prompting experimenters to confront ‘the questions we should ask of almost every human enterprise that intends to alter society: what is the purpose; who will be hurt; who benefits; and how can we know?’ (Jasanoff 2003, p. 240).

Interpreting technologies as themselves experimental provides a powerful way to reimagine the uncertainties and stakes of geoengineering research and explore the politics of geoengineering experiments themselves. There is a risk that this reframing nebulises the issues to the point of meaninglessness. I would argue that, with reference to the history and philosophy of experimentation we instead gain a new foothold on governance through close attention to the demarcation of what is considered certain or uncertain and stable or unstable within experimental systems.

For Hans-Jorg Rheinberger (1997), ‘experimental systems’ are a site for negotiation between the known and unknown. Experiments involve controlled surprises: ‘Experimentation, as a machine for making the future, has to engender unexpected events’ (ibid, pp.32–33). An experiment is made of two parts: the well-understood ‘technical objects’, and the ‘epistemic things’, which are the subject of inquiry. Following Rheinberger’s analysis, we can start to investigate the politics of experimentation through analysis of the bounding of certainties and uncertainties. We can ask, for example, what surprises are permitted in experimental systems and how uncertainties are imagined, understood and controlled in the construction of experiments.

## Opening up the ‘Surprise Room’

Beneath the now well-established conclusion of Science and Technology Studies (Nelkin 1979) that research in general expands rather than reduces the scope of uncertainty, we can analyse the strategic construction of uncertainty as a central part of scientific work (Wynne 1987). The notion of ‘surprise’ gives this work a harder political edge. Scientists themselves are not uncomfortable with the idea of surprise. The surprises that mark apparent ‘breakthroughs’ are central to scientific mythology. It is notable, for example, that psychologist Walter Mischel (2014) called his psychology laboratory at Stanford University’s crèche the ‘surprise room’. Gross (2010) makes the point that surprises, so integral to scientific novelty, nevertheless lie beyond conventional, containable categories of risk and probability. In this way, surprise is a useful lens on society’s relationship with scientific uncertainty. The precautionary critique of technological risk assessment relates at least in part to the inability of regulation to anticipate or deal with the unexpected. The surprising nature of technological risk is often a function of previous wilful ignorance as, for example, with asbestos, whose risks were anticipated, but ignored, more than a century before they were effectively controlled (EEA 2001). Rather than being concerned about surprises per se, therefore, constructivist analyses should be interested in questions of who defines, prepares for and responds to surprises, and how and why they do so.

Experiments play important performative, public and technological roles. Habermas (quoted in Radder 2009) argues that experimentation turns science into ‘anticipated technology’. Experiments involve the ‘systematic production of novelty…making and displaying new worlds’ (Pickstone 2001, p. 13; 30). The wider political importance of experimentation means that, when they take place in public, they are typically displays of certainty rather than genuine surprise (Shapin and Schaffer 1985; Collins 1988). As Collins puts it,

Where possible, experiments are still done in private because, the initiated aside, confidence in ‘the facts’ will not survive a confrontation with Nature’s recalcitrance. Only demonstrations or displays are gladly revealed for public consumption. (Collins 1988, p. 727)

A focus on experimental systems allows a reconsideration of the politics of geoengineering research. We can first reconsider, as others have begun to do, the inevitable experimentality of any future geoengineering technologies. This enables a focus on the contingency of technological promises that are currently offered as stable and certain. On this descriptive basis, we can secondly engage more normatively with the social aspects of ‘scientific’ experimentation. Seeing geoengineering as itself an experimental system allows for new, constructive insights into the governance of geoengineering experiments themselves.

## Geoengineering as Planetary Experiment

The problem to which geoengineering purports to offer a solution—climate change—has itself acquired a discourse of experimentalism. As scientific explanations of anthropogenic global warming were developed over the 20th Century, prominent scientific and political figures spoke of climate change as ‘a grand experiment’ (Guy Stewart Callendar), ‘a large scale geophysical experiment’ (Roger Revelle) or ‘a massive experiment with the system of this planet itself’ (Margaret Thatcher), emphasising both the profundity and uncertainty of humanity’s disruption to the climate system. For many climate scientists, the language of experimentation was a justification for the urgent development of scientific knowledge. Stephen Schneider argued in his book, *Laboratory Earth*, that ‘much of what we do to the environment is an experiment with Planet Earth, whether we like it or not’ (Schneider 1997, p. xiv). Schneider’s call-to-arms is issued to both policymakers and scientists: ‘It is no longer acceptable simply to learn by doing. When the laboratory is the Earth, we need to anticipate the outcome of our global-scale experiments before we perform them’, (Schneider 1997, p.xii).

Some technological enthusiasts, such as Stewart Brand, have used the description of climate change as a messy experiment as a rationale for controlled experimentation through geoengineering (e.g. Brand 2010). Schneider, in the few years before his death in 2010, took the opposite view, also shared by Al Gore (2009), who argued that ‘We are already involved in a massive unplanned planetary experiment… We should not begin yet another’ (through geoengineering). Most contemporary geoengineering researchers would agree that the scale of surprises generated by doing geoengineering would be too profound to be currently tolerable, although their views would vary on the global climate conditions that would make such risks worth taking.

The recent renewal of enthusiasm for geoengineering is at least partly due to Paul Crutzen, a Nobel Laureate atmospheric scientist who published a prominent paper (Crutzen 2006) arguing that scientists and policymakers should cautiously reconsider the idea of stratospheric particle injection, which had fallen out of fashion as attention to climate change mitigation had grown. Following Crutzen’s interjection, assessments of geoengineering proposals have sought to explore and explain the possible implications and uncertainties of deployment. Alan Robock (2008) provided an account of ‘20 reasons why geoengineering may be a bad idea’. The possible side effects Robock identifies range from environmental (the effects on local weather and continued acidification of oceans) and sociotechnical (the potential for lock-in to bad and irreversible technological systems and the impossibility of global consensus on the ideal temperature for the ‘global thermostat’) to the economic (high, escalating and uncertain costs) and political (the potential for militarisation of geoengineering technologies and the moral hazard that this technological insurance would introduce into delicate negotiations on mitigating climate change).

In 2008, the Royal Society began a study to respond to and inform the growing debate on geoengineering. Defining geoengineering as the ‘deliberate and large-scale intervention in the Earth’s climatic system with the aim of reducing global warming’, their report drew on a wide range of expertise, including social science, philosophy and law. As well as performing technical analyses of risk, cost, feasibility and speed across a wide range of geoengineering proposals, the Society’s report discussed questions of ethics and governance, noting that ‘The acceptability of geoengineering will be determined as much by social, legal and political issues as by scientific and technical factors’ (Royal Society 2009, p. ix). The uncertainties of geoengineering were foregrounded in parts of the report while in other parts the approach tends towards cost-benefit analysis. With explicit reference to Collingridge’s dilemma of control, the report described the possibility of technological lock-in contributing to shaping the future of geoengineering. Coming at a time when researchers were beginning to conduct experiments with ocean iron fertilisation, the Society was faced with calls to govern experimentation, particularly when experiments crossed borders between jurisdictions or took place in international waters (see Stilgoe 2015).

Although Robock’s assessment is broad, including ethical and political considerations, his sense of geoengineering-as-experiment is largely a technical one. He and colleagues (Robock et al. 2010) have argued that testing of geoengineering would be impossible without its full-scale deployment, in part because the signal of a response to any geoengineering would get lost in the noise of a chaotic climate system. Other geoengineering researchers have countered that, with careful scaling up and variation, the effects of geoengineering could be tested at a less than planetary scale (see MacMynowski et al. 2011). Even if this were to be true, the absence of either a hermetically-sealed scalable laboratory or a control run would blur any line drawn between research and deployment. Even without knowing what the technologies of an eventual geoengineering system would look like, sociologists of technology might agree that, as with the missiles studied by Donald Mackenzie (1993), they would be impossible to test except through use. Given the vast uncertainties within the climate system, any deployment of geoengineering, even at full scale, would be necessarily experimental, if not cybernetic (Jarvis and Leedal 2012). And when we consider whether these experiments might be in any way publicly credible, we bring climate models and their public contingencies into the apparatus too.

Further dimensions of the experimentality of geoengineering have been elucidated by Macnaghten and Szerszynski (2013) and Hulme (2014). For Mike Hulme, the experimentality of geoengineering relates to its outcomes being ‘unknown and unknowable.’ (Hulme 2014, p. 92). Using public focus groups, Macnaghten and Szerszynski (2013) explore the ‘social constitution’ of current geoengineering proposals. They point to public scepticism about the predictability of geoengineering and unearth profound public concerns that ‘pervasive experimentality will be part of the new human condition’ (Macnaghten and Szerszynski 2013, p. 470). (An earlier public dialogue exercise on geoengineering had been titled ‘Experiment Earth’ (Corner et al. 2011), reflecting similar concerns). Hulme (2014) joins Robock et al. in claiming that ‘The only experimental method for adequately testing system-wide response [to geoengineering] is to subject the planet itself to the treatment’. But Hulme’s argument is that this would also be an existential experiment on the human condition and humanity’s ability to govern. The geoengineered world Hulme anticipates would be necessarily totalitarian. In a similar vein, Szerszynski et al. (2013) have pointed to the potential for solar geoengineering to be incompatible with democratic governance as we know it. (Rayner (2014) has critiqued this analysis of geoengineering’s ‘social constitution’ on the grounds that it prematurely identifies the essence of technology that remains hugely uncertain).

The few NGOs that have begun campaigning against geoengineering were quick to adopt the language of experimentalism. One campaign, Hands Off Mother Earth (HOME), has the slogan ‘Our home is not a laboratory’. It is tempting to read geoengineering as the archetype of the whole world becoming a laboratory (Latour 1999), but this global view risks detachment from more immediate concerns. Describing the experimentality of geoengineering should not be considered mere speculation on implications (following Nordmann’s critique described above). Instead, by considering the contested boundaries of experiments (Davies 2010), we can engage with an emerging debate on the legitimacy of geoengineering experiments that are currently proposed or underway within and outside laboratories.

## Governing Geoengineering Experiments

The debate generated by the SPICE experiment reveals the politics of experimentation in geoengineering—the things that are held to be certain, the things regarded as uncertain and worthy of investigation and the things regarded as out-of-bounds. The conventional story, relayed by the science press, scientists and science funders, is that SPICE was a failure of governance and a failed experiment. Reading it as a social experiment, we can see that SPICE reveals a huge amount about what is at stake in geoengineering research.

Before SPICE, scientists had sought to establish a safe space for experimental research and a means of containment for the spiralling social and ethical questions that geoengineering had begun to generate. Following the publication of its report on geoengineering, the Royal Society initiated a Solar Radiation Management Governance Initiative that, among other things, became a forum for negotiation of the Society’s recommended ‘*de minimis* standard for regulation of research’ (Royal Society 2009, p. xii). Although some geoengineering researchers were eager to begin conducting experiments to elucidate the implications of geoengineering, including experiments that would intentionally perturb the environment in order to study it, SRMGI was unable to agree where or whether such a line should be drawn. Around the same time, environmental experiments involving ocean iron fertilisation and cloud aerosols seemed to encroach into the geoengineering issue but whose motivations were either obfuscated or explicitly directed at conventional environmental science (see Buck 2014; Russell 2012).

The cancellation of the SPICE experiment did not quell this discussion. Indeed, it may have intensified scientists’ attempts to identify and cordon off an area of no concern. Lawyer Edward Parson joined David Keith (2013) in arguing in *Science* for experimental thresholds. Their suggestion was that, above a certain upper limit (where there is a discernable effect on the environment), there should be a ban on geoengineering experiments. They also suggested a lower limit, beneath which experiments should be allowed to take place. Robock proposed an indoor/outdoor divide (Robock 2012), based on the premise that indoor activities are ethically justifiable while activities outside the laboratory demand additional scrutiny.

Victor and colleagues (2013) agree that ‘the key is to draw a sharp line between studies that are small enough to avoid any noticeable or durable impact on the climate or weather and those that are larger and, accordingly, carry larger risks’ (see also Parson and Ernst 2013). A report from the US Congressional Research Service talks of the need for a ‘threshold for oversight’ (Bracmort and Lattanzio 2013).

SPICE illustrates the trouble with such arguments. The reframing of the experiment as at least partly social challenges the attempt to hermetically seal it from public scrutiny. The SPICE scientists recognised this transition more vividly than anyone. One put it like this:

People want to draw a bright line… and say everything above it is legitimate and everything below it is dangerous and requires governance. But that [laughs] that attitude undermines everything that SPICE is trying to figure out, everything that SPICE has been challenged to do in terms of looking towards the far field, thinking about things like lock-in.

(Interview with SPICE scientist, quoted in Stilgoe 2015)

It is notable that the controversy generated by SPICE took place as much within the scientific community as around it. The idea of outdoor experimentation had already raised concerns among climate scientists. Raymond Pierrehumbert, a prominent climate scientist and critic of geoengineering, argued that,

The whole idea of geoengineering is so crazy and would lead to such bad consequences, it really is pretty pointless. We already know enough about sulfate albedo engineering to know it would put the world in a really precarious state. Field experiments are really a dangerous step on the way to deployment, and I have a lot of doubts what would actually be learned.^2^

David Keith had already argued that, ‘Taking a few years to have some of the debate happen is healthier than rushing ahead with an experiment. There are lots of experiments you might do which would tell you lots and would themselves have trivial environmental impact: but they have non-trivial implications’.^3^ In a BBC interview, he took issue with SPICE:

I personally never understood the point of that experiment. That experiment’s sole goal is to find a technocratic way to make it a little cheaper to get materials into the stratosphere. And the one problem we *don’t* have is that this is too expensive. All the problems with SRM are about who controls it and what the environmental risks are, not how much it costs. It’s already cheap. So from my point of view, I thought that was a very misguided way to start experimentation. 4

For Keith and other geoengineering researchers, an additional, thinly-disguised concern was that negative reactions to SPICE would threaten subsequent research and experimentation on geoengineering. The concern was not that SPICE represented a perturbative experiment that fell on the wrong side of the various thresholds under discussion—all agreed that the experiment was benign in terms of its direct environmental impact—but rather that it challenged a dominant sense of what was considered ‘well-ordered science’ (Kitcher 2003). SPICE was controversial not just because it was a prominent open-air experiment in a highly contested domain of technoscience, but also because it suggested an alternative demarcation of certainties and uncertainties.

Scientists’ responses to the SPICE proposal point to competing framings of the experimental system. Before SPICE, priority research questions for geoengineering were overwhelmingly concerned with *episteme* (knowing that) rather than *techne* (knowing how) (See Ryle 1971; Hansson 2014a, b). Hansson (2014b) has argued that experiments can blend *episteme* and *techne,* which provides an additional layer of explanation for the SPICE controversy. Scientists have, at least in the area of Solar Radiation Management, been reticent to openly explore the engineering constraints associated with creating a workable technology, instead reifying technological proposals dating back to the 1970s (e.g. Budyko 1974) and asking about the *impacts* of operationalising such ideas. SPICE brought engineers together with climatologists and atmospheric chemists, which had the effect of reconstructing the uncertainties considered relevant. Things previously considered stable, such as the cost and feasibility of stratospheric geoengineering, were treated as empirical questions. In Rheinberger’s language, technical objects became epistemic things, disrupting an implicit sense of the experimental system. The new possibilities of surprise generated by SPICE challenged the deterministic story of geoengineering.

The public nature of the SPICE experiment, and the subsequent debate it generated, created an opportunity for what Nerlich and Jaspal call ‘frame shifting’ (Nerlich and Jaspal 2012, p. 132). The initial assumption within the SPICE team was that the public would be interested, in a positive sense, or that the experiment could be a spur for a necessary debate on the ethics of geoengineering. Insofar as the experiment itself was problematised, the imagined public were those people within the immediate vicinity of the balloon who might bear witness to its launch. As the controversy unfolded, it became clear to the scientists and engineers that a relevant ‘public’ would not be so easily bounded. If we presume that the ‘slippery slope’ from research to deployment is completely frictionless, the relevant public could, as NGOs critical of SPICE implied, expand to encompass the world’s population.

The idea of ‘care’ implied by this reframing goes some way beyond the Royal Society’s idea of ‘carefully planned and executed experiments’ (Royal Society 2009, p. ix), which would include ‘Small/medium scale research (e.g. pilot experiments and field trials)’ (Royal Society 2009, p. 61). The Royal Society recognised that ‘Just as field trials of genetically modified crops were disrupted by some NGOs, it is foreseeable that similar actions might be aimed at geoengineering experiments involving the deliberate release of sulphate or iron (for example) into the air and oceans’. (Royal Society 2009, p. 15).

As a first step towards regulation, the Society argued for a international voluntary code of conduct, adding that ‘only experiments with effects that would in aggregate exceed some agreed minimum (de miminis) level would need to be subject to such regulation’ (Royal Society 2009, p. 52) (see also Bellamy 2014). In emphasising scientific self-governance and a scientific definition of contentious experimentation, they offer, in effect, to ‘take care’ of this issue, on behalf of society. This is ‘care’ in the paternalistic rather than democratic sense of the word.

Ralph Cicerone, who would go on to become president of the National Academy of Sciences, the Royal Society’s US equivalent, had argued at the time of Crutzen’s intervention that geoengineering research should be ‘considered separately from actual implementation… We should proceed as we would for any other scientific problem, at least for theoretical and modeling studies’ (Cicerone 2006). But if we are concerned with the sociotechnical imaginaries of geoengineering (Jasanoff and Kim 2009), then public, open-air experimentation may not be uniquely problematic especially if, as with SPICE, there is a consensus that such experimentation does not pose direct risks. The experimentality of geoengineering, exacerbated by the trajectory that its emergence and scale-up would follow, make problematic any attempt to draw a line between research and deployment.

Solar geoengineering began as a set of thought experiments, substantially inspired by the natural experiment of a massive volcanic eruption. Since the re-emergence as a topic of scientific research, there have been almost no substantial solar geoengineering experiments taking place in the open environment, with the ecosystem as part of the apparatus.^5^ SPICE was notable in that it became an in foro public experiment even in absence of an actual in situ trial taking place. However there have been a number of in silico experiments on general circulation models of the climate, whose results have informed the geoengineering debate. Ken Caldeira, another leading geoengineering researcher, has described in magazine interviews how he set out to demonstrate using computer models that geoengineering would be an unremittingly bad approach to global warming, but that he was taken aback by his own results. He told the New Yorker: ‘much to my surprise, it [geoengineering] seemed to work and work well’ (Specter 2012). (It is notable that these experiments *with* computer models are also experiments *on* the computer models (Schiaffonati, this issue; Stilgoe 2015). Given the power of such experiments to shape the promises and expectations of geoengineering, we might ask whether research inside the lab, involving computer models should self-evidently be free from public oversight or if there is a legitimate role for democratisation here too.

## From Noun to Verb

In the short time since geoengineering was rehabilitated as a legitimate area of scientific study, it has rapidly acquired a deterministic frame. Geoengineering has become naturalised by the scientists, social scientists, philosophers and others who have begun to focus on it. This has the effect of closing down governance discussions and absolving scientists of responsibility for fashioning this nascent sociotechnical imaginary. Imagining the potential for constructive governance of geoengineering and geoengineering research requires challenging this frame. I have suggested in this paper that focussing on the experimentality of geoengineering as an emerging technology provides one way forward. Rather than presuming a regime of technoscientific promises, I suggest instead that we rethink geoengineering within a regime of collective experimentation (Joly et al. 2010).

The table below (Table 1) summarises what such a reframing would mean in thought and practice. The first feature is a grammatical one. The regime of technological promises tends to reify geoengineering as a technology that is, if not already in the world, inevitable. This is an outcome of what Joly calls the ‘naturalisation of technological advance’ (Joly et al. 2010). Rather than treat the word as a noun (a gerund to be more precise) we can instead read ‘geoengineering’ as a verb (a present participle). This shift from noun to verb turns geoengineering from an object of governance (Owen 2014) to a work in progress, with all of the attendant uncertainties and poorly-defined responsibilities of those—scientists, engineers, philosophers, social scientists and others—implicated in the project.

**Table 1:** Two governance regimes for geoengineering research

	Regime of technoscientific promises	Regime of collective experimentation
‘Geoengineering’	…as noun	…as verb
Theory of technology	Instrumentalism	Substantivism/critical theory (see Feenberg 1999)
Responsibilities of researchers (including social scientists, philosophers etc.)	Assessment of technologies	Implicated in realising futures
Role of social science (see Macnaghten and Szerszynski 2013)	Proposing implications	Interrogating trajectories
Approach to uncertainty	Uncertainties seen as soluble through further research	Uncertainty seen as contested, inevitable and expanding
Approach to ethics	Speculative ethics and technology assessment	‘Technology accompaniment’ (see Verbeek 2010)
Characterising problems	‘Solutionism’, in which problems are assumed rather than explored	Reflexive approaches to problem identification and definition
Construction of public concerns	Technological development and perturbative experimentation	Open-ended, but may include imaginaries
Relationship between research and use	Scientific research is divorced from technological deployment	Research and deployment are entangled in the same social experiment
View of scientific autonomy	Negative liberty—Freedom *from*. (The ‘right to research’ viewed in libertarian terms)	Positive liberty—Freedom *to.* (The ‘right to research’ viewed in republican terms) (Brown and Guston 2009)
Relevant uncertainties	Implications of geoengineering	Implications, costs, feasibility, design
Governing experiments	Creating a ‘safe space’ for research	Engaging with entanglements
Experimental systems	Bounded by science	Including publics, politics, ecosystems and scientists themselves

The implications of this way of thinking can be seen if we consider new proposals for geoengineering ‘field experiments’. Keith and colleagues (2014) have recently described a suite of imagined experiments with which to explore the risks of further geoengineering research. Including the SPICE balloon experiment in their list, they imagine tests ranging from what they call ‘process studies’ up to ‘climate response’. Among these sits Keith’s own proposed SCoPEx experiment, which would take place in the lower stratosphere in order to test possible effects of solar geoengineering on stratospheric ozone. He has argued with colleagues that such experiments are ‘a necessary complement to laboratory experiments if we are to reliably and comprehensively quantify the reactions and dynamics defining the risks and efficacy of SRM’ (Dykema et al. 2014).

Keith and colleagues (2014) emphasise that the SCoPEx experiment would, along with most others in the list, generate negligible ‘radiative forcing’ (an intended cooling effect on the climate). SCoPEx would have climatic effects that are ‘small compared with that of a single flight of a commercial transport aircraft’. From their assessment, even larger ‘field experiments could be done with perturbations to radiative forcing that are negligible in comparison to the natural variability of climate at a global scale’ (Keith et al. 2014).

Taking geoengineering as a noun, one can see the rationale for such experiments, and for a governance regime that seeks to delimit regulation according to whether experiments are seen as posing direct climatic risks, at large scales and for extended periods of time, as Keith and colleagues suggest. Within this frame, the inclination is to bound the experimental system tightly, to reduce what what Keith and colleagues (2014) call ‘spurious disagreements’. The underlying motivation is to create a ‘safe space’ for research.

However, if we see geoengineering as a verb, under a regime of collective experimentation, things become less straightforward. Rather than prioritising freedom *from* experimental regulation, we might instead consider freedom in a positive sense, as a social licence to experiment. In addition to evaluating likely experimental risks and scales, we might also encourage scrutiny of experimental intentions and the imaginaries that sit behind them. Once we understand, as the SPICE scientists themselves did, that concerns with that experiment related to more than its direct risks, we can reframe other proposed experiments. This is not to presume that such experiments should therefore face additional governance from the top down. Indeed we would not wish those involved in experimentation and innovation to anticipate every possible future, not least because their activities are explicitly aiming to enable alternative and therefore unpredictable futures. The aim should instead be to experiment with experimentation, inviting further consideration of who should be involved in the definition and conduct of experiments. In practice, this may mean that geoengineering field experiments adopt the inter- and multi-disciplinary approaches that have started to take hold in other areas of geoengineering research (Szerszynski and Galarraga 2013).

The ambivalence of scientists and the political uncertainties surrounding geoengineering have meant that social scientists have been among those invited into various novel experimental collaborations (cf Rabinow and Bennett 2012; Stilgoe 2012). These interactions typically involve the renegotiation of what is considered known and unknown as parties try to break out of the mould that is cast for them by others. Perhaps the social scientists and others that have become part of the apparatus of geoengineering research can contribute to the realisation of an alternative vision, one of collective experimentation.

## Footnotes

1 See ‘Responsible Research and Innovation’, European Commission http://ec.europa.eu/programmes/horizon2020/en/h2020-section/responsible-research-innovation, accessed 1 Nov 2014; ‘Framework for responsible innovation, ESR, http://www.epsrc.ac.uk/research/framework/, accessed 1 Nov 2014; National Science Foundation (2004) International Dialog on Responsible Research and Development of Nanotechnology, http://www.nsf.gov/crssprgm/nano/activities/dialog.jsp, accessed 1 Nov 2014.

2 Quoted in Rotman, D. A Cheap and Easy Plan to Stop Global Warming, MIT Technology Review, February 8, 2013. http://www.technologyreview.com/featuredstory/511016/a-cheap-and-easy-plan-to-stop-global-warming/, accessed 19 December 2013

3 Quoted in The Economist, Lift Off, 4 November 2010, http://www.economist.com/node/17414216, accessed 19 December 2013.

4 Interviewed on BBC Hard Talk, BBC News Channel, 4:30AM Mon, 14 Nov 2011.

5 The only possible exception might be the E-PEACE experiment, which tested cloud formation off the pacific US coast in 2011, but this was not initially framed explicitly as a geoengineering test.
